# Impact of Thermal Processing on the Selected Biological Activities of Ginger Rhizome—A Review

**DOI:** 10.3390/molecules28010412

**Published:** 2023-01-03

**Authors:** Justyna Zagórska, Lidia Czernicka-Boś, Wirginia Kukula-Koch, Katarzyna Iłowiecka, Wojciech Koch

**Affiliations:** 1 Department of Food and Nutrition, Medical University of Lublin, 4a Chodzki Str., 20-093 Lublin, Poland; 2 Department of Pharmacognosy with Medical Plants Garden, Medical University of Lublin, 1 Chodzki Str., 20-093 Lublin, Poland

**Keywords:** *Zingiber officinale* Rosc., thermal treatment, transformation of components, biological activity, antioxidants

## Abstract

Ginger (*Zingiber officinale* Rosc.) is a spice, medicinal and cosmetic plant that has been known for centuries. It can be used in dried, fresh, marinated or candied form, and is also an essential ingredient in well-known curry blends. Ginger rhizomes are often freeze-dried as the first step in the preparation of the raw material. Many studies have proved that the composition and biological activity of ginger changes due to thermal processing. Therefore, the aim of the review was to summarize the scientific results on the impact of traditional and unconventional methods of the heat treatment of ginger rhizomes and their influence on the antioxidant and other selected biological activities of the plant. The review of the available scientific data is inconclusive, and it is hard to state unequivocally whether the thermal treatment of the raw material increases or decreases biological activity. Based on the presented literature review, it can be concluded that traditional cooking and microwave processing in general decrease the antioxidant activity of the ginger rhizome, whereas frying, autoclaving, blanching or traditional drying in the sun mostly lead to a significant increase in ginger activity. Interesting data were presented in the works describing the freeze-drying process during which the antioxidant potential of ginger increased.

## 1. Introduction

Spices and herbs have been known to mankind for centuries. They were valuable raw materials for barter trade, components of medicines and magical potions and ingredients for food. The first documented evidence of spices’ application dates back to around 1500 BCE. Ebers papyri denote the applications of cinnamon, saffron and anise [1,2,3]. Currently, spices are most often used for culinary purposes, as they improve the taste, color and aroma of drinks and dishes, and have a food-preserving effect [4]. However, an increasing popularity of their application for medicinal purposes can be observed. Spices and plant material may exhibit anti-inflammatory [5], antioxidant [6], anticancer [7], antibacterial [8] or blood glucose lowering properties [1,4], which encourages the use of them in foods and the design of new products from the group of functional foods, which could, in addition to the nutritional value, also have a preventive effect in relation to the development of non-communicable diseases (NCDs).

According to the World Health Organization, about two-thirds of the world’s population living in underdeveloped and developing countries use herbal medicines for their basic medical needs [3]. This is due to their general availability, low production cost and evidence on their effectiveness elaborated by traditional medical canons. The currently available studies confirm their pharmacological properties and bioactive content. Among them, alkaloids, tannins, phenolic compounds, vitamins, micro- and macro-elements regulate physiological functions to the highest extent [9]. These compounds are called secondary metabolites and are produced by plants during their growth to protect them against pathogens, herbivores or attracting insects. In addition, some of them have found application in medicine as drug components or matrices for the design of synthetic drugs [10]. The most popular spices include: oregano, basil, cumin, coriander, ginger, turmeric, garlic, cinnamon, cloves and black pepper [11,12].

Despite numerous studies on the pharmacological significance of plants, the actual role of herbs and spices in the treatment and prevention of various diseases, including chronic ones, is still not fully understood. Since most of these plants are commercially available and often processed to prolong their shelf-life, improve taste, texture, color and facilitate digestive processes [13], it is important to consider the effect of heat treatment on their chemical composition and hence on their pharmacological activity.

Among the most common methods of culinary processing, we can distinguish boiling in water, steaming, grilling, stewing, frying, drying, baking or stir-frying, described and characterized by the authors in their previous work [14]. Having in mind a multitude of processing techniques that can be used for the preparation of foods or herbs for consumption, the aim of the review is to set together the information on their impacts on the biological properties of ginger. 

Ginger (*Zingiber officinale* Rosc.) is a spice, medicinal and cosmetic plant known for centuries. The pharmaceutical raw material is the rhizome out of which polar or less polar extracts are obtained that contain either polyphenolic constituents or volatiles. The plant belongs to the Zingiberaceae family and was initially grown in Southeast Asia. Thanks to its refreshing aroma and spicy, pungent taste, it is a frequent additive to various types of dishes. It can be used in dried, fresh, marinated or candied form, and is also an essential ingredient in a well-known curry blend. Ginger is often used to flavor soups, sauces, meats, and is also added to fizzy drinks, teas and cakes [15,16,17]. In addition to its application in cuisine, the plant exhibits numerous healing properties, which were described in traditional medical systems, for example in Ayurvedic, Chinese and Unani medicine as an analgesic, digestive and anti-inflammatory drug [16,18]. The available literature data indicate a wide range of pharmacological activities of ginger, such as: antioxidant [19,20], anti-inflammatory [5,21], anticancer [7] and antibacterial [20] properties, in the inhibiting of nausea and vomiting in chemotherapy [22], in the treatment of diabetes and obesity [23] and in cardiovascular diseases [21]. In addition, current scientific reports indicate the possibility of using ginger in the prevention of neurodegenerative disease [24,25]. 

The beneficial pharmacological properties of ginger result from the presence of numerous bioactive compounds in its rhizome, which are also responsible for its characteristic taste and smell. Among the most important groups of chemical compounds there are polyphenols: gingerols, shogaols, paradols and their derivatives and terpene compounds divided into monoterpenes (camphor, β-phellandrene, geranial, neral, linalool) and sesquiterpenes (β-bisabolene, α-curcumene, zingiberene, α-farnesene and β-sesquiphellandrene) [26,27,28]. 

The presence of thermally unstable components from the group of terpenes and easily dehydrated phenolic components makes it important to study the effect of thermal treatment on their content, which was summarized in the authors’ previous work [14], but also on their biological properties.

**Figure 1:**
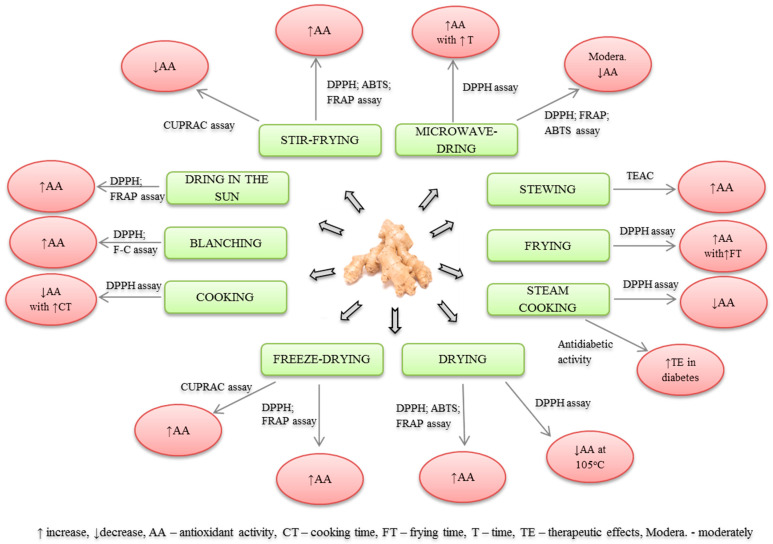
Selected types of thermal treatment and their effect on the activity of ginger rhizome.

This paper evaluates the effects of different thermal processing techniques on the selected biological activities of ginger extracts. The schematic outline of the content discussed in the review is presented in the Figure 1. 

### 1.1. Pharmacokinetics and Bioavailability of Ginger Compounds

Given the numerous pharmacological properties of ginger and its widespread use, little is still known about the bioavailability of its main compounds after thermal processing. The existing studies mainly describe in vivo tests in rodents after the oral or intraperitoneal administration of the whole extract or isolated compounds. These works demonstrate the rapid absorption and metabolism of ginger components in animals and humans, with the liver being the major organ that removes 6-gingerol [29]. Ginger compounds including 6-gingerol, 8-gingerol, 10-gingerol and 6-shogaol are detected in the plasma as glucuronide and sulfate conjugates. Both gingerols and shogaols showed similar absorption times in the circulatory system depending on their structure [18,28,30,31]. Zhang et al. [18] emphasized the effect of the length of the alkyl side chain on the reduced permeability of gingerols and shogaols to the blood and noted that shogaols have a longer residence time in the body than gingerols, which may have an impact on their higher biological activity. Other work by Jiang et al. [29] also investigated the tissue distribution of 6-gingerol after oral administration. The results indicated the highest concentration of this compound in the gastrointestinal tract, which justifies the application of 6-gingerol in digestive tract disorders. To show a systemic effect, active compounds from ginger must penetrate the blood and tissues, which is influenced by many factors, starting from the way the product is cultured and going through its metabolism in the digestive system. Due to their structure, gingerols and their analogues show poor solubility in water, which in turn affects their bioavailability [24]. The advancement of technology has contributed to the implementation of new solutions increasing the bioavailability of ginger compounds, consisting of the development of new transport systems such as: liposomes, nanoparticles, emulsions or micelles [32]. However, most often ginger, or its preparations, is consumed with the diet, and its composition and thus nutritional and physiological effect largely depends on the method of processing and thermal treatment, which is why it is so important to determine the optimal conditions for these processes.

### 1.2. Antioxidant Activity/Capacity of Ginger Compounds

Many studies have reported the numerous beneficial pharmacological effects of ginger, most of these data having been verified through experimental studies. However, the effect of heat treatment which in most cases ginger is subjected to during the preparation of meals and in industrial conditions, on its antioxidant capacity has not been well summarized. Therefore, the purpose of this non-limiting paper is to compare selected thermal treatment methods for the antioxidant activity of ginger rhizomes.

Most of the works dealing with the antioxidant topic of ginger extracts use in vitro tests, which save time and reduce research costs. Mainly, these methods can be divided depending on the reaction mechanism for: HAT (hydrogen atom transfer) consisting in the transfer of a hydrogen atom (free radicals are quenched by hydrogen donation), and the SET mechanism (single electron transfer) in which a single electron is transferred to the reduction of any compound. The aforementioned mechanisms may occur simultaneously, but one of them is always dominant. The methods following the HAT mechanism include: ORAC—oxygen radical absorbance capacity and TRAP—total radical trapping antioxidant parameter. On the other hand, the SET mechanism is used in the following methods: TEAC (Trolox Equivalent Antioxidant Capacity), FRAP (Ferric Reducing Antioxidant Power), CUPRAC (Cupric Reducing Antioxidant Capacity), using the Folina–Ciocalteau reagent with the ABTS (2,2’-azinobis (3-ethylbenzothiazoline-6-sulfonic acid) reagent and the DPPH (2,2-diphenyl-1-picrylhydrazyl) radical [33,34].

The TEAC method is used to determine the antioxidant potential in terms of the number of trolox equivalents, a derivative of vitamin E, per unit of mass of the tested sample [34].

The FRAP method is based on the reduction of the TPTZ (4,6-tri (2-pyridyl)-1,3,5-triazine) reagent by an antioxidant, producing an intense blue color with a maximum absorbance at 593 nm. The FRAP unit is assumed to be the ability to reduce 1 mole of iron (III) to iron (II) [34].

In the CUPRAC (Cupric Reducing Antioxidant Capacity) method, the measurement of the antioxidant capacity of the sample is based on the reduction of Cu(II) ions to Cu(I) bound in a complex with batocuproin (2,9-dimethyl-4,7-diphenyl-1,10-phenanthroline) or neocuproin (2,9-dimethyl-1,10-phenanthroline). The absorption maximum for the orange-yellow Cu(I) complex with neocuproin is 450 nm. Trolox or ascorbic acid are usually used as standards [35].

The Folin–Ciocalteu (F-C) method determines the total phenolics content (TPC). It consists in the reaction of polyphenolic compounds with a reagent, which is a mixture of sodium tungstate, sodium molybdate, lithium sulfate, bromine water and concentrated hydrochloric and phosphoric acids. In the reaction mechanism, an electron is transferred and a blue color is produced, the intensity of which is measured spectrophotometrically at a wavelength of 760 nm. The condition of the reaction is to provide an alkaline environment, adequate incubation time and temperature. The recommended reference standard is gallic acid and results are reported as milligrams gallic acid equivalents/100 g product [36].

The ABTS reagent method determines the degree of antioxidant scavenging of the ABTS radicals formed in reaction with, for example, potassium persulfate. The radical produced has an intense blue-green color that fades away, depending on the content of the antioxidants in the solution The course of the reaction is measured spectrophotometrically at a wavelength of 734 nm, and the antioxidant content is expressed in Trolox equivalents [37].

The method with the DPPH radical is based on the measurement of the ability of antioxidants to reduce the DPPH radical. As a result of the reaction, the radical captures an electron from the antioxidant and its dark purple color changes to yellow; the color change of the mixture is measured spectrophotometrically at a wavelength of 515 nm. The results are presented as percentages of inhibition or as the value of the IC_50_ parameter, which determines the concentration of the antioxidant, which causes a 50% decrease in the initial concentration of the radical. In addition, the result can be reported as the amount of Trolox reference substance equivalents or vitamin C [38].

## 2. Materials and Methods

A systematic search of the literature was conducted in the available online databases: Pubmed, Scopus, Web of Science, Science Direct, as well as in Google Scholar from 2004 to 2022. The search keywords were: “ginger”, “*Zingiber officinale*”, “thermal treatment”, “activity”, “cook”, “steam”, “drying”, “boil”, “blanch”, “microwave”, “fry”, “roast”, “freeze-drying” and “lyophilization”. The literature search was limited to the English language and articles related to the raw *Zingiber officinale* rhizome. In addition, the search results were checked individually by two authors. 

## 3. Introduction to thermal processing techniques

### 3.1. Cooking

#### 3.1.1. Traditional Cooking

In the case of cooking the ginger rhizome, the antioxidant activity decreased with increasing the duration of the thermal treatment at 40 °C and 98 °C. At both temperatures, the malondialdehyde (MDA) concentration was significantly increased; after 10 min of heating, the increase was about 5 mg MDA/kg ginger, and after 30 min, about 9 mg MDA/kg ginger. On the other hand, during the treatment at 70 °C, a reverse trend and an increase in antioxidant activity were observed [39]. In the studies of Koch et al. [40], the negative influence of cooking the ginger rhizome on its anti-radical potential was also presented. Along with the longer cooking time, the IC_50_ of the extract increased, which indicated a decrease in its antioxidant activity. After the longest tested cooking time of 15 min, the IC_50_ was 340 ± 80 µg/mL, while for fresh ginger it was at the level of 210 ± 10 µg/mL.

#### 3.1.2. Stewing 

In turn, studies by Chohan et al. [41] on stewing ginger indicate an increase in the antioxidant potential of the extract obtained from it. The Trolox equivalent antioxidant capacity assay (TEAC) of the ginger control extract was 4.6 ± 0.5 µmol/g, while after heat treatment it has significantly increased, up to 19.3 ± 1.1 µmol/g [41].

### 3.2. Frying

#### 3.2.1. Traditional Cooking

Koch et al. [40], revealed that extending the frying time of ginger without the addition of oil in a ceramic pan resulted in a significant decrease in the ginger antiradical activity, expressed as the IC_50_ value. The IC_50_ values for extracts from ginger fried for a specified time were as follows: 300 ± 22 µg/mL (2 min), 480 ± 23 µg/mL (5 min), 500 ± 26 µg/mL (10 min) and 940 ± 36 µg/mL (15 min). This study found that the longer ginger was cooked, the more its antioxidant activity decreased. It was shown that the free radical scavenging potential of the extract obtained from ginger fried for 15 min was more than four times lower than that of the fresh ginger extract (210 ± 10 µg / ml). The significant reduction of the antioxidant activity of the sample resulting from the heat treatment may be due to the dehydration and/or oxidation of the phenols present in the ginger, which results, for example, in the transition of gingerols to shogaols, i.e., compounds with a lower antiradical potential [42]. 

#### 3.2.2. Stir-Frying

In the research of Li et al. [43], stir-frying of the ginger rhizome was conducted in order to evaluate the antioxidant activity of the processed samples. Surprisingly, the obtained results revealed that the extract obtained from such processed ginger was characterized by a stronger antioxidant effect than the fresh ginger extract. The antioxidant activity of ginger after heat treatment increased as follows: from 64.84% to 77.22% (measure as the percentage of the deactivated free radicals in the DPPH test), 53.48% to 72.11% (ABTS) and from 0.46 Mmol/L FeSO_4_ to 0.68 Mmol/L FeSO_4_ (FRAP). 

#### 3.2.3. Blanching

The blanching process of the ginger was performed by Kumar et al. [44] during the production of ginger candies, whose antioxidant properties were than evaluated and compared to candies containing fresh product. The conducted processing increased the antioxidant potential of these candies compared to candies containing unblanched ginger. The antioxidant potential of the blanched product increased by 3.15%, which may be due to the increase in total phenolics. 

### 3.3. Steaming (Hot Vapor Processing)

Interesting results were obtained in the work of Namm et al. [45], where the antidiabetic properties of ginger rhizomes were investigated. Steaming the ginger has a positive effect on its potential to prevent and treat diabetes. It was proved that the antidiabetic effect of ginger is mainly due to the presence of 1-dehydro-6-gingerdione, which blocks the K_ATP_ (ATP-sensitive potassium channel) channel in β-pancreatic cells and thus stimulates insulin secretion. The increase in antidiabetic activity after treatment may be due to the fact that the extract from steamed ginger has a higher content of 1-dehydro-6-gingerdione, compared to the unprocessed ginger extract. For the regeneration of pancreatic islets at the level of 50%, steamed ginger extract with a concentration of 0.3 µg/mL was enough, while in the case of the unprocessed extract, it was necessary to use as much as a 33 times higher concentration (9.9 µg/mL) to achieve the same effect. 

In the studies by Takahashi et al. [46], the antifungal activity of the ginger oil obtained after steaming the rhizome was studied. No significant differences between oils from fresh and processed rhizomes were noticed. The essential oils obtained from fresh and steamed ginger showed the same anti-Candida activity. For both oils, the concentration inhibiting filament formation in 50% (IC_50_) was 12.5 µg/mL, and the IC_90_ was 50 µg/mL.

The effect of steaming on the antioxidant activity of ginger was also investigated using the DPPH radical method. The results of the study performed by Teng et al. [47] showed that the antioxidant activity of the processed raw material extract decreased, but the difference was insignificant (99.69 and 99.73%, for processed and fresh rhizome, respectively). 

Opposite conclusions were obtained in the study by Koch et al. [40]. The authors revealed that treating ginger with hot steam increased the antioxidant activity of ginger. The greatest increase in antioxidant activity in relation to the activity of the fresh ginger extract was recorded for ginger autoclaved for 60 min. The IC_50_ for the fresh ginger extract was 210 ± 10 µg/mL, while for the extract subjected to hot vapor processing for 60 min it changed to 160 ± 16 µg/mL. The lower the IC_50_ value, the higher the antioxidant activity. Therefore, it was proved that the steaming of the ginger rhizome resulted in a significant increase in its antioxidant activity.

### 3.4. Drying

#### 3.4.1. Drying in the Sun

The air-drying of ginger had a positive effect on the anti-Candida activity of the essential oil obtained from the processed material. Takahashi et al. [46] revealed that the minimum oil concentration that inhibits fungal filament formation by 50% (IC_50_) was the same for both fresh and processed ginger oil (12.5 µg/mL). On the other hand, the IC_90_ for the oil obtained from the dried ginger was 25 µg/mL and was two times lower than the IC_90_ for fresh ginger oil. These data clearly indicate that the oil from a processed plant had significantly stronger antifungal activity in comparison to the oil obtained from a fresh ginger rhizome. Also, the minimum inhibitory concentration (MIC) for fresh ginger oil (400 µg/mL) was twice as high as the MIC for dried ginger oil (200 µg/mL) (RPMI medium/medium-Roswell Park Memorial Institute). The degree of Candida growth inhibition at 4000 µg/mL was similar for both oils (medium/YPG medium). For fresh ginger oil it was 98.0%, and for dried ginger oil 97.5%. In turn, the IC_50_ value for the first oil was 2 µg/mL, and for the second one 1 µg/mL (YPG medium/vehicle). This also proves a stronger inhibition of fungal growth by processed ginger oil. Amoah et al. [48] revealed that the microbial load of the ginger extract differs depending on the degree of processing of the rhizome. In the case of fresh ginger, the fungus load was 6.00 × 103 CFU/g, and in the sun-dried ginger extract it was 5.00 × 103 CFU/g. In turn, the bacterial loads were 30.0 × 109 CFU/g and 1.10 × 109 CFU/g for the oil from a fresh and dried plant, respectively. This demonstrates the stronger antimicrobial activity of the oil obtained from the processed ginger rhizome. 

In the work presented by Chumroenphat et al. [49], the effect of thermal treatment on the antioxidant properties of ginger was investigated. Drying the ginger rhizomes in the sun had a positive effect on their antioxidant activity. In the DPPH test, the antioxidant activity of the extract was 32.28 and 42.11% for 9- and 12-month-old fresh rhizomes, respectively. After drying in the sun, it increased to 62.93 and 64.23%, respectively. In turn, in the FRAP method the values for fresh ginger were 90.20 and 106.12 µmol Fe (II)/g dry weight (DW), respectively. In turn, for the extracts from processed rhizomes 462.91 and 387.23 µmol Fe (II)/g DW, for 9- and 12-month-old plant material, respectively. Gümüşay et al. [50] used the CUPRAC test to evaluate the influence of thermal processing on the antioxidant activity of the ginger rhizomes. The antioxidant potential of the sun-dried ginger extract was lower than that of the fresh ginger extract. The obtained data for the fresh ginger extract were 2176.13 mg Trolox/100 g DW, and for the sun-dried ginger extract almost two times lower: 1220.616 mg Trolox/100 g DW.

#### 3.4.2. Other Drying Methods

In the research of Amoah et al. [48], the microbiological activity of ginger was investigated under the influence of various drying techniques: a stainless steel solar dryer and drying in a concrete tent-type solar dryer. In the first study, the fresh ginger extract was characterized by a microbiological load of 30.0 × 109 CFU/g of bacteria and 6.00 × 103 CFU/g of fungi. In turn, in the extract obtained from ginger stainless steel solar-dried, these values were, respectively, 3.00 × 108 CFU/g of bacteria and 3.00 × 102 CFU/g of fungi, which demonstrates the stronger antimicrobial and antifungal potential of the thermally processed ginger. In the second method, the extract obtained from ginger dried in a concrete solar dryer showed stronger antibacterial and antifungal activity, in comparison to the fresh ginger extract. The microbiological load of the ginger dried in this way significantly decreased to 5.1 × 107 CFU/g of bacteria and 3.00 × 103 CFU/g of fungi.

Jolad et al. [51] investigated the influence of drying on the anti-inflammatory activity of white and yellow ginger. It was revealed that for commercially dried white ginger, the anti-inflammatory activity measured as the concentration that inhibits LPS-induced PGE_2_ production (PGE_2_ IC_50_) was 0.055 µg/mL, which was similar to the concentration of fresh white ginger (0.051 µg/mL). Fresh yellow ginger showed a weaker anti-inflammatory effect as compared to other samples, because its PGE_2_ IC_50_ concentration was 0.072 µg/mL. It was also proved that drying had no effect on the cytotoxicity of the ginger rhizome. For dried and fresh yellow ginger, the cytotoxic dose was the same: 50 µg/mL, while for fresh white ginger it was five times lower (10 µg/mL). 

Various studies investigated the influence of heating in different conditions on the antioxidant activity of the ginger rhizome and its products. In a study by Tiwari et al. [52], it was found that heating at 120 °C for 1 h adversely affected the antioxidant potential of both ginger powder and essential oil. The antioxidant activity of the heated powder in relation to the unprocessed ginger decreased from 8.46 to 6.16% (against the standard, BHT-Butylated hydroxytoluene) and from 3.19 to 1.63% (against the standard, Pyrogallol). However, in the case of essential oil, the activity after heat treatment decreased from 1.54 to 1.35%. 

In the study by Koch et al. [40] on antioxidant activity, it was found that after drying the ginger rhizome for 2 h at 105 °C the antioxidant activity significantly decreased in comparison to the fresh plant. In the DPPH test, the concentration that deactivated half of the free radicals in the sample (IC_50_) for fresh ginger was 210 µg/mL, and for dried 300 µg/mL. In turn, Li et al. [43] investigated the antioxidant activity of ginger extracts dried at 40 °C using the DPPH, ABTS and FRAP methods. They revealed that the extract from dried ginger showed a much stronger antioxidant effect, confirmed by all the methods, than the fresh ginger extract. The ability to neutralize the DPPH radical for the fresh ginger extract was at the level of 64.84%, while for the dried ginger extract it was 90.12%. In the ABTS test, the antioxidant activity was 53.48 and 88.44%, for fresh and processed ginger rhizome, respectively. The ferric reducing antioxidant power (FRAP) method revealed that the antioxidant activity of the fresh extract was 0.46 Mmol/L FeSO_4_, and for the extract from a processed plant it was almost two times higher, 0.89 Mmol/L FeSO_4_.

#### 3.4.3. Oven and Hot-Air Drying

Research on the influence of drying on the antioxidant properties of ginger was also conducted by An et al. [53]. It was revealed that drying ginger with hot air in an oven adversely affected its antioxidant activity. Using the DPPH method and ascorbic acid (AA) as the reference compound, AEAC (Ascorbic Acid Equivalent Antioxidant Capacity), it was shown that the antioxidant activity of the fresh ginger extract was at the level of 3.49 mg AA/g DW (IC_50_ 1.11 mg/mL extract), while for hot air-dried ginger extract 3.37 mg AA/g DW (IC_50_ 1.15 mg/mL extract). On the other hand, the FRAP and ABTS values were 19.37 mg Vc/g DW (vitamin C equivalents) and 64.45 mg Trolox/g DW and 17.41 mg Vc/g DW and 62.22 mg Trolox/g DW, for the fresh and dried ginger extract, respectively. It suggests that the technique used to evaluate the antioxidant activity of the fresh and processed plant may also have a significant influence on the final result. This may also partially explain the opposite outcomes obtained by the different authors on a similar subject. 

In the work by Chumroenphat et al. [49], the aspect of the influence of drying on the antioxidant activity of ginger was also taken into account. The extracts obtained from oven-dried ginger (40–70 °C) had a stronger free-radical-scavenging potential than the fresh ginger extracts. The strongest antioxidant activity tested by the DPPH and FRAP methods was demonstrated by ginger extracts dried in an oven at 60 °C. Using the DPPH method, an increase in the antioxidant activity of the extracts from 9- and 12-month-old rhizomes was noted, from 32.28 and 42.11% (fresh ginger) to 78.45 and 79.34% (ginger dried in an oven at 60 °C). The FRAP method confirmed the significantly higher activity of the ginger extract dried at 60 °C in comparison to a fresh plant. For the 9-month-old ginger extract, after heat treatment, the value increased from 90.20 to 650.45 µmol Fe(II)/g DW. In turn, for 12-month-old rhizomes the increase was from 106.12 to 662.31 µmol Fe(II)/g DW.

Gümüşay et al. [50], received other results; as a result of drying the ginger rhizome in the vacuum oven (at 60 °C, 0.025 mbar, 36 h), its antioxidant activity significantly decreased. The CUPRAC values for the fresh ginger extract were at the level of 2176.13 mg Trolox/100 g DW, while as a result of the thermal treatment they decreased to 1470.043 mg Trolox/100 g DW. 

In another study, the effect of the oven heating of ginger at 100 °C for various periods of time on the antioxidant activity of the extract was investigated. It was revealed that thermal treatment significantly reduced the antioxidant activity of the plant. In the case of unprocessed ginger, the activity was 31.8 µmol Trolox eq./g, while after the treatment for 1, 3 or 6 h it was in the range of 25.5–26.9 µmol Trolox eq./g. It was concluded that the heat treatment may decompose some of the components of ginger or transformed them into compounds with lower antioxidant activity. In turn, the activity of the scavenging superoxide radicals (2’dG method) after individual heating periods was as follows: 53.3 (0 h-fresh), 50.8 (1 h), 58.8 (3 h) and 56.1 (6 h) (µmol Trolox eq./g), which proved that at the beginning it decreased, but later (with a prolonged time of heating) it increased [54]. Such discrepancies may be explained because in the initial stage of heating some of the antioxidant compounds of ginger are transformed into compounds with lower activity, e.g., gingerols into shogaols. However, extending the heating time may contribute to an increase in the degree of extraction due to the destruction of cell walls and the easier release of active components, which results in an increase in the antioxidant activity of the extract.

#### 3.4.4. Blanching as Pretreatment and Drying in the Drying Chamber

In another study, the effect of blanching and drying the ginger rhizome on the change of antioxidant activity was assessed. The antiradical potential of the extracts was determined as % scavenging activity using the DPPH and ABTS methods. For the fresh ginger extract, these values were at the levels of 86.67 (DPPH) and 47.28% (ABTS). After 2 min blanching, the antioxidant activity decreased to 81.14 and 42.39% in the DPPH and ABTS tests, respectively. In the same study, the effect of drying the ginger samples in a drying chamber on its antiradical properties was also evaluated. An increase in the antiradical potential (measured in a DPPH test) of the ginger extracts subjected to blanching (as a pretreatment) and then dried at 60, 70 or 80 °C was observed. The activity of the samples dried only at the tested temperatures and subjected to a two-stage treatment was at a similar level for all extracts and amounted to approx 87%. Similar results were obtained using the ABTS method, where the % scavenging activity value was similar for dried and blanched and then dried samples and ranged from 53.93 to 56.14%. Summarizing these data, it can be concluded that blanching as the only applied thermal treatment weakens the antioxidant activity of ginger, while drying itself (regardless of temperature) or combined with blanching has a positive effect on its antiradical scavenging activity [55].

### 3.5. Microwaves

The available literature includes studies evaluating the effect of heating the ginger rhizome with the use of microwave radiation on the antioxidant activity. In the work by Koch et al. [40], the positive effect of microwaves on the antioxidant activity of the ginger rhizome was revealed using the DPPH test. The longer the plant material was exposed to microwaves, the more active the extract obtained from it was. The IC_50_ decreased from 210 µg/mL (for fresh ginger) to 150 µg/mL (for ginger heated with microwaves for 5 min), which proved a significant increase in the antioxidant potential of the ginger rhizome processed using microwaves.

In another study, the exposure of the ginger extract to microwave radiation for 1 min resulted in a decrease in its antioxidant activity tested by DPPH assay. After thermal treatment using microwaves, the IC_50_ increased from 201.81 to 353.73 ppm, which proved a significant reduction of the antioxidant activity of the rhizome [56].

Similar conclusions were obtained in the study by An et al. [53], in which the use of microwave radiation in the drying process showed a negative effect on its antioxidant activity. The study was conducted using various tests to evaluate the antioxidant properties of the samples. The AEAC and IC_50_ values for the fresh ginger extract were 3.49 mg AA/g DW and 1.11 mg/mL, and for the microwave-dried ginger extract 3.42 mg AA/g DW and 1.13 mg/mL. The FRAP value as a result of the heat treatment decreased from 19.37 to 15.66 mg Vc/g DW. The results obtained using the ABTS method also revealed the decrease in antioxidant activity after thermal processing using microwave radiation. For the fresh ginger extract, the antioxidant activity was 64.45 mg Trolox eq./g DW, and for the microwave-dried extract 60.06 mg Trolox eq./g DW.

#### Intermittent Microwave-Convection Drying

In the study by An et al. [53], the influence of the heat treatment of ginger with intermittent microwave-convection drying on the antioxidant potential was analyzed. Activity measurements were expressed as AEAC, IC_50_, FRAP and ABTS values. For the fresh ginger extract, the above-mentioned values were as follows: 3.49 mg AA/g DW, 1.11 mg/mL of extract, 19.37 g Vc/g DW and 64.45 mg Trolox eq./g DW. However, for the dried ginger extract they were 3.58 mg AA/g DW, 1.08 mg/mL of extract, 21.91 g Vc/g DW and 71.68 mg Trolox/g DW. The obtained results revealed that in the majority of tests the antioxidant activity of the ginger increased after microwave-convection drying. It is also worth emphasizing that, based on the tests used to evaluate the antioxidant activity of the sample, inconclusive results might be obtained.

### 3.6. Carbonization

Li et al. [43] evaluated the antioxidant activity of the ginger rhizome subjected to carbonization. The results of the study showed that the extract obtained from such processed ginger was characterized by higher antioxidant properties in comparison to the extract obtained from fresh ginger. The antioxidant activity of the extracts was tested by three methods, each of which confirmed the stronger effect of the extract from heat-treated ginger. The fresh ginger extract neutralized 64.84% of the DPPH free radicals, while the processed ginger extract 70.03%. In the ABTS test, the value of the first extract was 53.48%, and the value of the second extract was 65.80%. In turn, the ability to reduce iron (III) ions for the fresh ginger extract was 0.46 Mmol/L FeSO_4_, and for the carbonized ginger extract 0.57 Mmol/L FeSO_4_.

The data presented in the study have been collected and summarized in Table 1 and Table 2.

### 3.7. Other

In addition to the conventional methods of the thermal processing of the ginger rhizome, the available research also uses unconventional methods that require advanced equipment: freeze-drying and infrared.

#### 3.7.1. Freeze-Dried/Lyophilization

Chumroenphat et al. [49] investigated whether the freeze-drying of ginger increases the antioxidant activity of the ginger extract. The first test used to evaluate the antioxidant capacity was the DPPH method. The results showed that after treatment the activity of the extract increased from 32.28 to 66.34% in the case of 9-month-old rhizomes and from 42.11 to 67.35% for 12-month-old rhizomes. These results were also confirmed using the FRAP method. For 9-month-old rhizome extracts, the values of 90.20 µmol Fe (II)/g DW for the fresh ginger extract and 549.32 µmol Fe (II)/g DW for the lyophilized ginger were recorded. In turn, for 12-month-old rhizomes, this parameters were 106.12 and 558.31 µmol Fe (II)/g DW for fresh and processed rhizomes, respectively. Similar conclusions from the research were obtained by Gümüşay et al. [50]. The freeze-drying process positively influenced the antioxidant potential of the ginger rhizomes. The CUPRAC values for the fresh ginger extract were 2176.13 mg Trolox eq./100 g DW, while for the freeze-dried ginger extract 2555.574 mg Trolox eq./100 g DW. The increase in the value of this parameter proves the beneficial effect of this method of processing ginger on its antioxidant activity. Also, the work of An et al. [53] confirms that freeze-drying of ginger increases its antioxidant potential. In this study, indices of antioxidant activity such as AEAC, IC_50_, FRAP and ABTS were determined for fresh and lyophilized ginger extract. The values for particular parameters in the case of fresh ginger extract were as follows 3.49 mg AA/g DW, IC_50_ 1.11 mg/mL of extract, 19.37 g Vc/g DW and 64.45 mg Trolox eq./g DW, while for the processed ginger extract 3.69 mg AA/g DW, 1.05 mg/mL of extract, 20.88 g Vc/g DW and 68.65 mg Trolox eq./g DW. Obtained results revealed that the freeze-dried ginger extract showed stronger antioxidant activity in comparison to the fresh ginger extract.

#### 3.7.2. Infrared Drying 

In the work of An et al. [53], another method of the non-conventional treatment of ginger was used—drying using infrared. The antioxidant potential was evaluated using three different techniques, the DPPH, FRAP and ABTS methods. The obtained results revealed that the infrared drying of ginger contributes to an increase in the antioxidant activity of the extract obtained from it. Using the first of the above-mentioned methods, the AEAC value was determined, which was 3.49 mg AA/g DW for the fresh ginger extract (IC_50_ 1.11 mg/mL extract), and for the processed ginger extract 3.55 mg AA/g DW (IC_50_ 1.09 mg/mL extract). In turn, the FRAP value for the tested extracts was 19.37 mg Vc/g DW (for the fresh ginger extract) and 22.14 Vc/g DW (for the dried ginger extract). In the third test, the increase in the antioxidant activity after thermal processing was also confirmed. The antioxidant potential expressed as ABTS for the unprocessed ginger extract was 64.45 mg Trolox eq./g DW, and for the treated ginger extract 66.79 mg Trolox eq./g DW. However, it should be emphasized that the differences were insignificant. 

The presented literature includes articles on a given issue, but their results differ significantly depending on the author. First of all, it should be remembered that often not a single compound, but a mixture of phytochemicals, is responsible for a particular biological activity. Additionally, there may be synergistic or additive effects between some of the components responsible for health benefits. The majority of the studies that evaluate the influence of thermal processing on the biological activity of the ginger rhizomes focus on the antioxidant activity of the processed plant. Only a few studies, which can be found in the scientific literature, evaluate other biological activities, such as antifungal or antidiabetic properties. Studies performed by Takashi et al. [46] or Nam et al. [45] suggest that thermally processed ginger (using superheated steam) retains or even increases its antidiabetic and antifungal activity. Drying ginger in the sun also increases its antifungal activity.

However, the majority of the studies assess the antioxidant activity of the thermally processed ginger rhizome. Determining the effect of water on antioxidant activity is a separate issue, since boiling in the presence of water is suspected to reduce it. The processes taking place during the operation of high temperature may have a detrimental effect on phytochemicals, including phenolic compounds, and cause their decomposition into compounds with a lower molecular weight and lower activity. However, in some studies, the antioxidant activity has increased, which may be due to changes in the intrinsic properties of the product matrix or to the destruction of the cellular wall and thus the increased penetration of the solvent during the extraction process. Changes in the biological activity of ginger as a result of thermal processes are a secondary process to the complicated changes in the composition of the raw material, which were extensively described and discussed in our previous review work [14]. It is difficult to say unequivocally whether the thermal treatment of the raw material increases or decreases biological activity. Based on the presented literature review, it can be concluded that traditional cooking, hot-air drying, oven-drying and microwave processing in general decrease the antioxidant activity of the ginger rhizome, whereas frying, autoclaving, blanching or traditional drying in the sun mostly lead to a significant increase in the ginger activity. 

The majority of the presented research suggests that traditional cooking and microwave drying most significantly decrease the antioxidant capacity of the ginger rhizome. This is caused mostly due to an induced degradation of the major active constituents of the ginger rhizome—gingerols, which are primarily responsible for its antioxidant activity. It was revealed that the microwave processing of ginger led to a significant decrease in gingerols such 6-, 8- and 10-gingerol. Also, in the analyzed studies the TPC and TFC (Total Flavonoid Content) parameters were alleviated [53]. According to Lim and Murtijay [57], the strong thermal degradation of phenolic compounds in ginger is mostly due to the rapid and intense increase in the operational temperature of the microwave source. It was also proposed that the rapid production of heat activates oxidative enzymes such as peroxidases and polyphenol oxidases, which leads to a decrease in polyphenol concentration and finally a decrease in the antioxidant activity of the plant material. Moreover, it was also proved that microwave treatment caused not only the loss of the phenolic fraction in ginger rhizomes, but also significantly reduced the content of essential oil, including oxygenated monoterpenes and other aliphatic compounds. What is interesting is that the losses of these compounds were the greater the higher the microwave energy used [58]. Moreover, it was revealed that various types of drying using hot air decreased the content of some of the main ingredients of ginger essential oil, such as zingiberene, geranial, β-sesquiphellandrene, α-curcumene and β-bisabolene and resulted in the formation of various esters, such as bornyl acetate and propanoic acid derivatives [53]. The long-term exposure of ginger to oxygen at high temperature could contribute to the synthesis of these compounds as a result of the esterification of the alcohols to esters [59]. It was also observed that the content of the 6-, 8- and 10- gingerols in the extract was reduced, whereas the 6-shogaol concentration increased. These changes were the most significant in oven-dried ginger, which is in agreement with the data presented in the current review, as this method of thermal processing was described as one of the most harmful to the antioxidant properties of the ginger rhizome. 

Similar conclusions can be drawn considering cooking. The available data suggest that the decrease in the antioxidant potential of the ginger rhizome due to steam cooking may be caused by a significant reduction of the 6-gingerol concentration, which is the most abundant phenolic compound in the plant and primarily responsible for its antioxidant properties. Thus, a significant reduction in its concentration may cause a strong reduction in its biological properties [47,60]. 

In general, it seems that thermal processing methods of ginger rhizome, such as various types of hot-air drying and cooking in which the raw material is subjected to high oxygen and water exposure, alleviate gingerol concentration, which results in a decrease in its biological properties, including antioxidant properties. Because the biological properties of the ginger rhizome result from a synergistic effect of various active components, the reduction in terpene concentration, observed especially during hot-air drying and cooking, may be an additional mechanism partially responsible for the loss of the antioxidant activity of the ginger rhizome [46,61,62]. 

On the other hand, traditional processing techniques such as frying or blanching lead to an increase in phenolic or volatile component concentration, which in turn leads to a significant increase in the biological properties of ginger [7,45,52]. 

Interesting data were presented in the works describing the freeze-drying process during which the antioxidant potential of ginger increased. Although it is a technique that requires advanced and expensive equipment, this may be a method introduced on a large scale in the future as the most effective way of processing ginger, allowing an increase in some of its biological activities. 

It should also be emphasized that many research results are inconclusive, which is related to the various techniques used for the evaluation of antioxidant activity in vitro (DPPH, ABTS, FRAP or CUPRAC), as well as the various modifications of these methods, e.g., using different antioxidant standards or the modified conditions of the performed experiments. These chemical methods used for the determination of antioxidant capacity are based on measuring the effects of substances with antioxidant properties on the rate of oxidation processes in the sample. It is difficult to characterize any of these methods as universal. Unfortunately, there is often no correlation between results obtained on the same test sample using different assays. In vitro assays are widely used, but the ability to predict in vivo activity from their results is questioned. This is because chemical methods do not take into account the physiological temperature and pH conditions, the impact of bioavailability and metabolic changes taking place in a living cell [57]. However, because such methods are cheap, fast and easy to perform, there are no better techniques for the initial screening of the antioxidant activity of plant material. 

A review of the research on the effect of treatments on the chemical composition and activity of ginger will allow for the optimization of the method of processing the rhizome in a way that allows the retention of active phytochemicals in the raw material, to obtain the highest biological activity of the product. The collected information will also allow the analysis of the processing processes not only in terms of phytochemical research, but also in terms of food safety aspects.

## 4. Conclusions and Perspectives

The aim of the study was to compile the available data on the impact of selected thermal processing methods on the selected biological activities of the ginger rhizome. Based on the collected information, it has been shown that the application of high temperature leads to changes in the biological activity of the plant, even if the gathered results are often inconclusive. Among the traditional thermal techniques of ginger processing, microwaves and cooking seem to be the most destructive to active components and thus reduce the biological activity of the rhizome to the highest degree. On the other hand, frying or blanching retain the biological activity of the plant, as the reported concentration of active constituents (phenolics and terpenes) was retained or even increased. From all of the methods discussed in the present review, freeze-drying seems to be the most effective technique of drying the ginger rhizome, as this method not only retained but significantly increased the antioxidant activity of the raw material.

It is worth emphasizing that the studies on thermal processing in other species from the Zingiberaceae family led to breakthrough discoveries. Studies conducted in recent years have confirmed that various methods of the thermal processing of turmeric cause changes in the content of the active constituents and thus affect its antioxidant and anticancer activity [52,63,64]. Dahmke et al. [65] confirmed that the traditional way of frying turmeric before consumption, practiced for centuries by Indians, leads to the formation of derivatives with an exceptionally high anticancer activity. Even if there are many studies regarding the influence of thermal processing on the changes in the composition and biological activity in the ginger rhizome, so far no discoveries have been made proving that the heat treatment of ginger rhizomes can contribute to the synthesis of completely new derivatives with increased biological activity. More studies are needed to evaluate if the thermal processing of ginger rhizomes may lead to the synthesis of new derivatives with higher biological activity, as was proved for other species from this family; however, the aim of this review paper is to draw attention to the subject of ginger processing and its impact on final activity. 

## Figures and Tables

**Table 1:** Effect of traditional processing on pharmacological activity of ginger *Zingiber officinale*.

Lp	Type of Processing	Processing Conditions	Tested Activity	Results	Reference
1	Cooking	10, 20 and 30 min cooking at 40, 70 and 98 °C	Antioxidant activity (MDA assay)	↑ or ↓ activity	[39]
2		5, 10 and 15 min boiling followed by diethyl ether extr.	Antioxidant activity (DPPH assay)	↓ activity with ↑ cooking time	[40]
3	Stewing	10 min boiling of GIN (1:100 *w/v*), followed by 1 h stewing, kept covered for 1 h and cooled	Antioxidant activity (TEAC)	↑ antioxidant activity	[41]
4	Frying	2, 5, 10 and 15 min frying in a ceramic pan followed by diethyl ether extr.	Antioxidant activity (DPPH assay)	↑ activity with ↑ frying time	[40]
5	Blanching	10 min blanching of dried GIN in boiling water with 2% citric acid followed by 60 min drying at 50 °C	Antioxidant activity of candies (DPPH and Folin assay)	↑ activity by ginger candies	[44]
6	Steam cooking	120 min steaming of GIN at 3 kgf/cm^3^ and 97 °C, followed by 40 h drying in oven at 45 °C and 5 h extr. with 70% ethanol at 70 °C	Antidiabetic activity	↑ therapeutic effects in diabetes	[45]
7		18 h steaming of GIN followed by freeze-drying at −70 °C for 7 days and 10 min microwave extr. at 180 W with 70% ethanol	Antioxidant activity (DPPH assay)	↓ activity	[47]
8	Steam heating	120 min heating followed by drying and EO extr.	Anti-*Candida* activity.	= activity	[46]
9	Autoclaving	30, 60 and 90 min heating at 128 °C and diethyl ether extr.	Antioxidant activity (DPPH assay)	↑ activity	[40]
10	Stir-frying	7 min frying of dried rhizomes (40 °C) mixed with sand (1:10 *w/w*) at 220 °C and extr. with methanol	Antioxidant activity (DPPH, ABTS, FRAP assays)	↑ activity	[43]
11	Microwave-drying	1, 3 and 5 min drying at 600 W and extr. with diethyl ether	Antioxidant activity (DPPH assay)	↑ activity with ↑ time	[40]
12		1 min drying of enzymatic extract with 10% DMSO at 700 W and 2.45 GHz	Antioxidant activity (DPPH assay)	↓ activity	[56]
13		Drying until 50% ↓ in GIN moisture at 5 w/g energy density followed by 1 w/g to dryness	Antioxidant activity (DPPH, FRAP, ABTS assays)	moderately ↓ activity	[53]
14	Oven heating	60 min drying of EO and powdered rhizome at 120 °C, followed by methanol: ethyl acetate 50:50 extr. (*v/v*)	Antioxidant activity (DPPH assay) of EO and powder	↓ activity in both	[52]
15		1, 3 or 6 h drying of GIN in 20% ethanol at 100 °C	Antioxidant activity (DPPH and peroxide scavenging assays)	↓ activity in DPPH assay = / slightly ↑ activity in peroxide test	[54]
16	Drying in the sun	7 days drying and EO extr.	*Candida* inhibition	↑ activity	[46]
17		Dried 9- and 12-months old GIN	Antioxidant activity (DPPH and FRAP assay)	↑ activity	[49]
18		72 h drying at 25–30 °C.	Antioxidant activity (CUPRAC assay)	↓ activity	[50]
19		Fresh ginger rhizome immersed for 1 h in a 10% vinegar solution, then dried for 3 days at a temperature of 25.5–36.6 °C	Antimicrobial (*Salmonella* sp.) and antifungal activity (*Aspergillus*, *Penicillium*, *Mucor* and *Rhizopus*)	↑ activity	[48]
20	Solar dryer	15 h drying of GIN, immersed for 1 h in 10% vinegar, at 44–58 °C	As above	↑ activity	[48]
21	Concrete tent-type solar dryer	5 days drying of GIN, immersed for 1 h in 10% vinegar, at 32–42 °C	As above	↑ activity	[48]
22	Drying	Commercially dried ginger powder extr. with dichloromethane	Anti-inflammatory activity (PGE_2_ assay)	= activity	[51]
23		2 h drying of GIN at 105 °C followed by diethyl ether extr.	Antioxidant activity (DPPH assay)	↓ activity	[40]
24		GIN drying at ca. 40 °C, methanol extr.	Antioxidant activity (DPPH, ABTS, FRAP assays)	↑ activity	[43]
25	Hot-air drying	GIN drying at 60 °C	Antioxidant activity (DPPH, FRAP, ABTS assays)	↓ activity	[53]
26	Oven drying	9- and 12-months GIN drying at 40 °C, 50 °C, 60 °C and 70 °C	Antioxidant activity (DPPH and FRAP assays)	↑ activity, best for 60 °C and 70 °C	[49]
27		36 h GIN drying at 60 °C	Antioxidant activity (CUPRAC assay)	↓ activity	[50]
28	Vacuum oven drying	36 h drying at 60 °C at 0.025 mbar	Antioxidant activity (CUPRAC assay)	↓ activity	[50]
29	Carbonization	Drying at ca. 40 ° C, followed by heating and frying until carbonization	Antioxidant activity (DPPH, ABTS, FRAP assays)	↑ activity	[43]
30	Intermittent microwave-convection drying	GIN drying with 700 W with hot air drying at 60 °C, using pulse regulation (PR) of first PR = 2 (5 s on–5 s off) up to 50% water content, and PR = 6 (5 s on–25 s off) to the end of drying.	Antioxidant activity (DPPH, FRAP, ABTS assays).	↑ activity	[53]
31	Blanching as pretreatment to drying chamber	2 min blanching with 95 ± 1 °C water and drying at 60, 70 or 80 °C with 0.3 m/s air velocity	Antioxidant activity (DPPH and ABTS method)	Blanching ↓ activity Air drying ↑ activity	[55]

↑—increased activity; ↓—decreased activity; = no effect on the activity; GIN—fresh ginger rhizomes; extr.—extraction; EO—essential oil.

**Table 2:** Effect of freeze-drying and infrared drying (unconventional methods) on pharmacological activity of ginger.

Lp	Type of Processing	Processing Conditions	Tested Activity	Results	Reference
1	Freeze-drying/Lyophilization	Freeze-drying of 9- and 12-months old at −40 °C	Antioxidant activity (DPPH and FRAP assays)	↑ activity	[49]
2		24 h freeze-drying of GIN −50 °C and 0.133 mbar	Antioxidant activity (CUPRAC assay).	↑ activity	[50]
3		12 h freezing of GIN at −40 °C followed by freeze-drying at 20 Pa, 25 °C and −58 °C of heating plate and cold trap, respectively	Antioxidant activity (DPPH, FRAP, ABTS assays)	↑ activity	[53]
4	Infrared drying	Drying of GIN by three red glass lamps (225 W each)	Antioxidant activity (DPPH, FRAP, ABTS assays)	↑ activity	[53]

↑—increased activity; GIN—fresh ginger rhizomes.

## Data Availability

Not applicable.
